# Development of one-step SYBR Green real-time RT-PCR for quantifying bovine viral diarrhea virus type-1 and its comparison with conventional RT-PCR

**DOI:** 10.1186/1743-422X-8-374

**Published:** 2011-07-29

**Authors:** Ni Zhang, Zhengwen Liu, Qunying Han, Jianming Qiu, Jinghong Chen, Guoyu Zhang, Zhu Li, Sai Lou, Na Li

**Affiliations:** 1 Department of Infectious Diseases, First Affiliated Hospital, School of Medicine, Xi'an Jiaotong University, Xi'an 710061, Shaanxi Province, the People's Republic of China; 2 Department of Microbiology, Molecular Genetics and Immunology, University of Kansas Medical Center, Kansas City, KS, USA; 3 Institute of Endemic Diseases, School of Medicine, Xi'an Jiaotong University, Key Laboratory of Environment and Genes related to Diseases, Ministry of Education, Xi'an 710061, Shaanxi Province, the People's Republic of China

**Keywords:** Bovine viral diarrhea virus type-1, cRNA standard, SYBR Green I RT-PCR, Quantitation, Cell culture

## Abstract

**Background:**

Bovine viral diarrhea virus (BVDV) is a worldwide pathogen in cattle and acts as a surrogate model for hepatitis C virus (HCV). One-step real-time fluorogenic quantitative reverse transcription polymerase chain reaction (RT-PCR) assay based on SYBR Green I dye has not been established for BVDV detection. This study aims to develop a quantitative one-step RT-PCR assay to detect BVDV type-1 in cell culture.

**Results:**

One-step quantitative SYBR Green I RT-PCR was developed by amplifying cDNA template from viral RNA and using *in vitro *transcribed BVDV RNA to establish a standard curve. The assay had a detection limit as low as 100 copies/ml of BVDV RNA, a reaction efficiency of 103.2%, a correlation coefficient (R^2^) of 0.995, and a maximum intra-assay CV of 2.63%. It was 10-fold more sensitive than conventional RT-PCR and can quantitatively detect BVDV RNA levels from 10-fold serial dilutions of titrated viruses containing a titer from 10^-1 ^to 10^-5 ^TCID_50_, without non-specific amplification. Melting curve analysis showed no primer-dimers and non-specific products.

**Conclusions:**

The one-step SYBR Green I RT-PCR is specific, sensitive and reproducible for the quantification of BVDV in cell culture. This one-step SYBR Green I RT-PCR strategy may be further optimized as a reliable assay for diagnosing and monitoring BVDV infection in animals. It may also be applied to evaluate candidate agents against HCV using BVDV cell culture model.

## Background

Bovine viral diarrhea virus (BVDV), the etiological agent of bovine viral diarrhea/mucosal disease, is a worldwide pathogen in cattle. Based on the characteristic of presence/absence of visual cytopathology in infected cells, BVDV has been segregated into two biotypes, cytopathic (CP) and noncytopathic (NCP). According to sequences from the 5'-nontranslated region (5'-NTR) of the viral genome, BVDV has been divided into two genotypes, types 1 and 2 [1,2]. BVDV infection results in diarrhea, acute and chronic mucosal disease, persistent infection and immunotolerance, immunosupression, pregnant cow abortion, dead fetus and abnormal fetus. Having not been controlled by classical vaccination, BVDV seriously endangers the cattle herds [1].

BVDV belongs to the genus *Pestivirus *of the family *Flaviviridae*, which also comprises the genera *Flavivirus *and *Hepacivirus *[3], and contains one single-stranded, plus-sense RNA genome of approximately 12.5 kb [4]. Hepatitis C virus (HCV) belongs to the same family *Flaviviridae *with BVDV and the genomes of HCV and BVDV both consist of a 5'-NTR, a single open reading frame and a 3'-nontranslated region (3'-NTR) [5]. Both viruses may cause chronic infections in their respective hosts. Thus, BVDV, especially the type 1 NADL strain, also acts as a surrogate model of HCV based on all these similarities and the feasibility to be cultured *in vitro *[6,7]. Therefore, a high-throughput assay for precise detection of BVDV was essential not only for the diagnosis and disease evaluation of BVDV infected animals but also for the screening of candidate anti-HCV agents in cell culture.

Serological assay such as enzyme-linked immunosorbent assay [8,9] and molecular biological methods such as conventional reverse transcription-polymerase chain reaction (RT-PCR) [10-12] and nested PCR [13] for the detection of BVDV have been developed. However, serological assay is usually time-consuming and the results are not very accurate and specific [14]. RT-PCR is sensitive and specific for the detection of BVDV, but the analysis of RT-PCR amplified fragment is usually followed by a procedure of agarose gel electrophoresis, which may result in contamination of amplified products in the laboratory. With the advent of real-time fluorogenic quantitative PCR (FQ-PCR), TaqMan-PCR for the detection of BVDV has been developed [15-17]. In comparison with TaqMan-PCR, SYBR Green PCR assay, a real-time FQ-PCR technique using SYBR Green I dye, has the advantages of being easy to design, relatively low setup and running costs [18] and possibly more precise results and linear decay plot [19]. Two-step SYBR Green I RT-PCR assay has been used to detect BVDV [20]. However, one-step real-time fluorogenic quantitative RT-PCR assay based on SYBR Green I dye has not been established for BVDV detection.

The present study developed a high-throughput one-step SYBR green I real-time quantitative RT-PCR assay for the detection of BVDV type 1 in cell culture and obtained the copy numbers of virus using a constructed RNA standard curve. The performance of the one-step SYBR Green I RT-PCR assay was also evaluated.

## Materials and methods

### Cells and virus

Primary calf testis (CT) cells were prepared from healthy newborn calf as described elsewhere [21]. Experimental procedures were approved by the Institutional Animal Care and Use Committee of Xi'an Jiaotong University.

Cytopathic BVDV (type 1, strain NADL, National Animal Disease Laboratory, Ames, Iowa, USA) was purchased from China Institute of Veterinary Drugs Control (Beijing, China) with the virus dilution in serum-free MEM.

### Virus titration

Cells at 80% confluence in a 96-well plate were infected with the virus at a series of dilutions from 10^-1 ^to 10^-9 ^with eight wells for each dilution, and were maintained at 37°C in 5% CO_2 _for 134 h. The titer of 50% tissue culture infective dose of virus (TCID_50_) was calculated with the method of Reed-muench [22].

### RNA isolation

CT cells incubated in 25 ml culture flask at 37°C in 5% CO_2 _and grown into 80% to 90% cell confluence were infected with BVDV and incubated for 110 h. When 100% CPEs was seen, the cells were frozen and thawed 3 times at -80°C, and then the supernatant was collected, clarified by centrifugation (1,000 × *g*) and stored at -80°C for RNA extraction. Viral RNA used for the construction of cRNA standards for BVDV RNA was extracted from the supernatants using the QIAamp viral RNA mini kit according to the manufacturer's instructions (QIAGEN China Co., Ltd. Shanghai, China). Viral RNAs from a series of dilutions from 10^2 ^TCID_50 _to 10^-5 ^TCID_50 _were also extracted for further experiment.

### Construction of cRNA standards for BVDV RNA

#### Primer design and modification for PCR

Referring to the BVDV sequence (GeneBank accession no. M31182), the primer pairs specific for the BVDV NS5B region were designed as 5'-ACACCAAAGCCTGGGACACT-3' (position 11226-11245 of the NADL sequence) and 5'-CTCCCTCTCTGCCCATTTCTT-3' (position 11386-11406 of the NADL sequence). The forward primer was modified with the incorporation of a T7-promoter sequence (5'-TAATACGACTCACTATAGGG-3') onto the 5'-end of the primer. The modification was essential for performing *in vitro *transcription with the T7 RNA polymerase followed. The modified forward primer and the reverse primer were used for the construction of cRNA standards. The primers were synthesized and purified by TaKaRa (TaKaRa Dalian Biotechnology Co., Ltd. Dalian, China). The RNA standards constructed had a size of 184 base (nt. 11226 to 11406 of BVDV sequence and 3 base T7-promoter sequence).

#### Amplification of cDNA template for *in vitro *transcription by RT-PCR

Viral RNA from cell cultures was taken for cDNA synthesis. RNA PCR Kit (AMV) (TaKaRa Dalian Biotechnology Co., Ltd. Dalian, China) was used for RT-PCR according to the manufacturer's instructions. Reverse transcription (RT) was performed in a final volume of 10 μl, containing 0.5 μl random 9 mers, 2 μl MgCl_2 _(2.5 mM), 1 μl 10 × RNA PCR buffer, 1 μl dNTP mixture, 0.25 μl RNase inhibitor, 0.5 μl AMV reverse transcriptase (5U/μl), 4 μl (3 μg) of viral RNA and 0.75 μl DEPC-treated H_2_O. RT was performed with the following program: 10 min at 30°C, 30 min at 42°C, 5 min at 99°C and 5 min at 5°C. PCR was performed in a total reaction volume of 50 μl reaction mixture by adding 40 μl of the mixture, containing 10 μl of 5 × PCR buffer, 28.75 μl of sterilized distilled water, 0.25 μl of TaKaRa Ex Taq™ HS and 0.5 μl of each primer (20 μM) as described above for constructing BVDV cRNA standards, into the tube containing 10 μl RT products. PCR was performed in a cycling condition as follows: 2 min at 94°C followed by 30 cycles of 30 sec at 94°C, 1 min at 55°C and 1 min at 72°C with a final step at 72°C for 3 min to allow complete extension of all amplified fragments. The amplified products had an expected size of 201 bp on 1% agarose gel electrophoresis.

#### *In vitro *transcription

PCR products amplified with the modified primer pairs were used as the template to synthesize complementary RNA (cRNA) by *in vitro *transcription with T7 RNA polymerase after being purified by ethanol precipitation. *In vitro *transcription T7 Kit (TaKaRa Dalian Biotechnology Co., Ltd. Dalian, China) was applied according to the manufacturer's protocol. The RNA standards obtained were stored at -80°C until use.

#### Quantification of the RNA standards

The OD value of RNA standard concentrations was measured at 260 nm/280 nm on Thermo Scientific NanoDrop™ 1000 Spectrophotometer (NanoDrop Technologies, LLC, Wilmington, DE, USA). The viral RNA genomic copy number of the RNA standards was calculated according to the following formula: RNA copy number (copies/μl) = RNA concentration (g/μl) × 6.02 × 10^23^/345 × RNA length (b).

### One-step quantitative real-time RT-PCR

The RNA standards were used to construct standard curves spanning 10^7^-10^2 ^copies/ml by 10-fold serial dilutions. One-step quantitative real-time RT-PCR (Q-RT-PCR) was performed by One-step SYBR^® ^PrimeScript™ RT-PCR Kit II (Perfect Real Time, TaKaRa Dalian Biotechnology Co., Ltd. Dalian, China) on Bio-Rad iQ5 Multicolor Real-Time PCR Detection System (170-9780, BIO-RAD Laboratories, Hercules, CA, USA). The forward primer and reverse primer were synthesized and purified by TaKaRa (TaKaRa Dalian Biotechnology Co., Ltd. Dalian, China), and the sequences were 5'-TGACACCATCACCGACCAC-3' (position 11323-11341 of the NADL sequence) and 5'-CTCCCTCTCTGCCCATTTCTT-3' (position 11386-11406 of the NADL sequence), respectively, amplifying a 84 bp fragment. Reverse transcription was carried out in a condition of 5 min at 42°C and 10 sec at 95°C, and PCR reaction was performed 40 cycles of 5 sec at 95°C and 30 sec at 60°C.

### Validation of reproducibility of one-step SYBR Green I RT-PCR assay

To assess the intra-assay and the inter-assay variability, RNA standards from 1 × 10^7 ^to 1 × 10^2 ^copies/ml and titrated viruses at different dilutions were tested by one-step SYBR Green I RT-PCR assay in triplicate in a single assay (intra-assay) and at three different days (inter-assay). The coefficient of variation (CV) of threshold cycle (Ct) was determined.

### Validation of specificity of one-step SYBR Green I RT-PCR assay

To differentiate specific from nonspecific amplified products, the amplified products obtained by one-step SYBR Green I RT-PCR assay were identified by analysis of melting peaks of BVDV RNA standards from 1 × 10^7 ^to 1 × 10^2 ^copies/ml and titrated viruses at different dilutions.

### Conventional RT-PCR

The forward primer and reverse primer with the same sequences as those used in one-step SYBR Green I RT-PCR assay were synthesized and purified by TaKaRa (TaKaRa Dalian Biotechnology Co., Ltd. Dalian, China). Viral RNA at a volume of 2 μl from titrated viruses at 10-fold serial dilutions was tested by RT-PCR with RNA PCR Kit (AMV) (TaKaRa Dalian Biotechnology Co., Ltd. Dalian, China) on MJ research PTC-200 peltier thermal cycler (APE-BridgePath Scientific, Frederick, MD, USA) according to the manufacturer's instructions. Complementary DNA was synthesized using viral RNA as template by reverse-transcriptase in a program consisted of 10 min at 30°C, 30 min at 42°C, 5 min at 99°C and 5 min at 5°C and subsequently amplified by PCR in the program including an initial denaturation step at 94°C for 5 min, followed by 40 cycles with denaturation at 94°C for 30 sec, annealing at 60°C for 30 sec and extension at 72°C for 45 sec, and a final extension at 72°C for 5 min.

### Comparisons of one-step SYBR Green I RT-PCR and conventional RT-PCR

Serial dilutions of the viral titrations detected by one-step SYBR Green I RT-PCR and conventional RT-PCR in parallel were compared. The amplified products were electrophoresed on 2.5% agarose gels and the optical density (OD) of the image was analyzed with UVP BioImaging Systems by LabWorks Image Acquisition and Analysis Software 4.0 (Ultra-Violet Products Ltd., Cambridge, UK).

### Statistical analysis

All data were analyzed using statistical software SPSS13.0 (SPSS Inc., Chicago, IL, USA). Inter-group comparison of OD values was analyzed by Student's t test with significance level α = 0.05.

## Results

### Complementary DNA template for in vitro transcription

**Figure 1:**
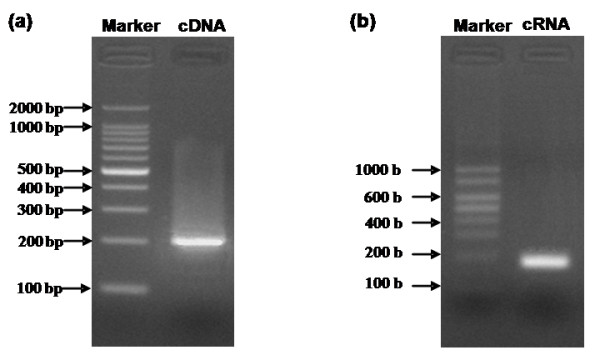
**Agarose gel electrophoresis of cDNA template (a) and cRNA standard (b)**. (a) 1% agarose gel electrophoresis of cDNA template synthesized by RT-PCR. Complementary DNA (cDNA) template with a length of 201 bp synthesized using viral RNA as template by reverse-transcriptase and subsequently amplified by PCR. Marker: 100 bp DNA Ladders. (b) 3% agarose gel electrophoresis of cRNA standard. Complementary RNA (cRNA) standard with a length of 184 b synthesized using the amplified cDNA as a template by *in vitro *transcription. Marker: RNA Marker RL1,000.

The amplification of viral RNA from cell cultures by RT-PCR successfully obtained the cDNA template for *in vitro *transcription with a size of 201 bp on 1% agarose gel (Figure 1a), which was in accordance with the expected size.

### Transcripts quantification of RNA standards

*In vitro *transcription with T7 RNA polymerase using the cDNA template successfully generated the cRNA transcripts, which had a size in accordance with the expected 184 b on 3% agarose gel (Figure 1b).

### Quantification of RNA standards

The concentration of RNA standards was quantified to be 205.6 (ng/μl) by spectrophotometer. The copy numbers of RNA standards were calculated to be 1.9 × 10^12 ^copies/μl according to the formula as described above.

### Complementary RNA standard curves

**Figure 2:**
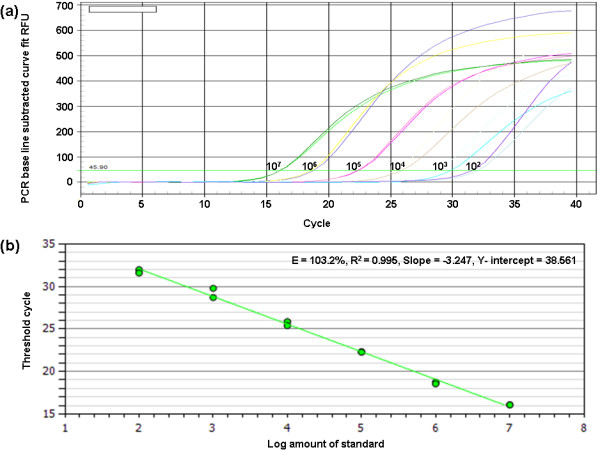
**BVDV RNA melting curves (a) and BVDV RNA standard curve (b)**. (a) BVDV RNA melting curves showing 10-fold serial dilutions of standard RNA from 10^7 ^to 10^2 ^copies/ml amplified by SYBR Green RT-PCR on Bio-Rad iQ5 Multicolor Real-Time PCR Detection System. (b) BVDV RNA standard curve produced by SYBR Green RT-PCR on Bio-Rad iQ5 Multicolor Real-Time PCR Detection System using 10-fold serial dilutions of standard RNA transcribed *in vitro *as standard templates.

SYBR Green I PCR amplifications were performed to established standard curves for BVDV RNA using the serially diluted RNA standards obtained by *in vitro *transcription. The detection and quantification limits were determined using Ct values obtained by six serial dilutions ranging from 10^7 ^to 10^2 ^copies/ml of the standard RNA (Figure 2a). The reaction efficiency of the assay using the slope (slope = -3.247) from the linear equation (copy number of the virus = 10^(Ct -38.561)/-3.247^) was estimated to be 103.2%. The correlation coefficient (R^2^) was 0.995 (Figure 2b).

### Reproducibility of one-step SYBR Green I RT-PCR assay

The intra-assay and inter-assay variations of one-step SYBR Green I RT-PCR assay were summarized in Table 1. The maximum CV in the intra-assay tests was 2.63%, demonstrating good reproducibility.

**Table 1:** Analytical reproducibility of one-step SYBR Green I RT-PCR assay in the inter- and intra- assay

	Levels of cRNA (copies/ml)						**TCID**_**50**_		
	**10**^**2**^	**10**^**3**^	**10**^**4**^	**10**^**5**^	**10**^**6**^	**10**^**7**^	**10**	**10**^**-2**^	**10**^**-5**^

The inter- assay									
Ct values (mean ± S.D.)	31.63 ± 0.276	29.31 ± 0.482	25.56 ± 0.344	22.19 ± 0.286	18.55 ± 0.299	15.95 ± 0.300	24.10 ± 0.065	26.71 ± 0.570	28.87 ± 0.308
CV (%)	0.87	1.64	1.34	1.28	1.61	1.88	0.27	2.13	1.06
The intra- assay									
Ct values (mean ± S.D.)	31.78 ± 0.274	29.25 ± 0.769	25.63 ± 0.327	22.30 ± 0.039	18.65 ± 0.142	16.08 ± 0.008	24.11 ± 0.046	26.71 ± 0.337	29.01 ± 0.266
CV (%)	0.86	2.63	1.27	0.17	0.76	0.05	0.19	1.26	0.91

### Specificity of one-step SYBR Green I RT-PCR assay

**Figure 3:**
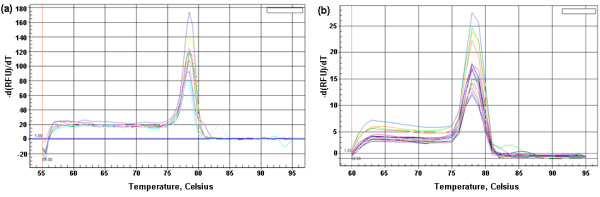
**Melting peaks analysis on the PCR products of SYBR Green RT-PCR on Bio-Rad iQ5 Multicolor Real-Time PCR Detection System**. (a) Melting peaks of PCR product from cRNA standards. (b) Melting peaks of PCR product from BVDV RNA of 10-fold serial dilutions of titrated virus.

Melting peaks analysis on the PCR products of cRNA standards showed that there was no primer-dimers and non-specific products and only a single peak was visible in the melting peak chart (Figure 3a). There was also no non-specific amplification observed from 10-fold serial dilutions of titrated virus known to be positive for BVDV (Figure 3b).

### Sensitivity comparison of one-step SYBR Green I RT-PCR with conventional RT-PCR

BVDV RNA in 10-fold serial dilutions of titrated virus from 10^2 ^TCID_50 _to 10^-5 ^TCID_50 _were detected by one-step SYBR Green I RT-PCR and conventional RT-PCR in parallel. BVDV RNA in titrated virus at 10^-5 ^TCID_50 _quantitatively detected by one-step SYBR Green I RT-PCR was equivalent to 8.79 (±1.18) × 10^2 ^copies/ml of BVDV RNA.

**Figure 4:**
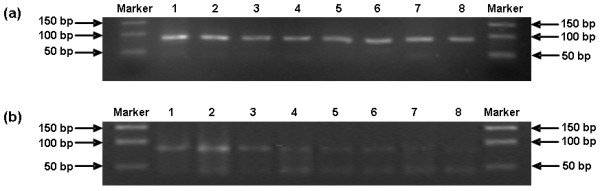
**Comparison of one-step SYBR Green I RT-PCR with conventional RT-PCR for dectecting BVDV RNA of titrated viruses at 10-fold serial dilutions**. (a) 2.5% agarose gel electrophoresis of the amplified products with a length of 84 bp obtained by one-step SYBR Green I RT-PCR. Marker: 50 bp DNA Marker, Lane 1: 100 TCID_50_, Lane 2: 10 TCID_50_, Lane 3: 1 TCID_50_, Lane 4: 10^-1 ^TCID_50_, Lane 5: 10^-2 ^TCID_50_, Lane 6: 10^-3 ^TCID_50_, Lane 7: 10^-4 ^TCID_50_, Lane 8: 10^-5 ^TCID_50_. (b) 2.5% agarose gel electrophoresis of the amplified products with a length of 84 bp obtained by conventional RT-PCR. Marker: DNA Marker DL 500, lane 1: 100 TCID_50_, lane 2: 10 TCID_50_, lane 3: 1 TCID_50_, lane 4: 10^-1 ^TCID_50_, lane 5: 10^-2 ^TCID_50_, lane 6: 10^-3 ^TCID_50_, lane 7: 10^-4 ^TCID_50_, lane 8: 10^-5 ^TCID_50_.

The detection limit of conventional RT-PCR was 10^-4 ^TCID_50_. The OD values of titrated virus at 10^-1 ^TCID_50_, 10^-2 ^TCID_50_, 10^-3 ^TCID_50 _and 10^-4 ^TCID_50 _were 1295.77 ± 127.06, 1263.33 ± 106.46, 1359.53 ± 80.14 and 1340.00 ± 11.09, respectively. There was no significant difference in OD values of PCR products by conventional RT-PCR between titrated virus at 10^-1 ^TCID_50 _and at 10^-4 ^TCID_50 _(*P *= 0.580), between titrated virus at 10^-2 ^TCID_50 _and at 10^-4 ^TCID_50 _(*P *= 0.283), and between titrated virus at 10^-3 ^TCID_50 _and at 10^-4 ^TCID_50 _(*P *= 0.697) (Figure 4b).

Compared with conventional RT-PCR assay, one-step SYBR Green I RT-PCR assay was 10-fold more sensitive and could be used to quantitatively detect the levels of BVDV RNA from 10-fold serial dilutions of titrated virus from 10^-1 ^TCID_50 _to 10^-5 ^TCID_50_, which were (9.67 ± 0.36) × 10^3 ^copies/ml, (4.54 ± 1.05) × 10^3 ^copies/ml, (2.64 ± 0.75) × 10^3 ^copies/ml, (1.37 ± 0.16) × 10^3 ^copies/ml and (8.79 ± 1.18) × 10^2 ^copies/ml, respectively (Figure 4a). There were significant differences in the levels of the virus RNA between titrated virus at 10^-1 ^TCID_50 _and at 10^-2 ^TCID_50 _(*P *= 0.001), between titrated virus at 10^-3 ^TCID_50 _and at 10^-4 ^TCID_50 _(*P *= 0.046), between titrated virus at 10^-4 ^TCID_50 _and at 10^-5 ^TCID_50 _(*P *= 0.014) except those between titrated virus at 10^-2 ^TCID_50 _and at 10^-3 ^TCID_50 _which showed a marginal significance (*P *= 0.063).

## Discussion

This study established CT cell culture system for BVDV proliferation. The specific primers for constructing cRNA standards and real-time RT-PCR were designed from the genomic sequence of the NS5B of BVDV genotype 1, NADL stain, whose sequence was different from that of BVDV genotype 2, resulting in characterization of BVDV type-1.

RNA standards in this study were generated using BVDV RNA in cell cultures by *in vitro *transcription with T7 RNA polymerase for quantifying BVDV RNA by real-time RT-PCR. A RNA standard curve with a linear range of 6 log units was set up and quantitation from 10^7 ^to 10^2 ^copies of the standard RNA were determined. The levels of RNA from samples for inspection were quantified by extrapolation of fluorescence signals against standard curves representing the initial copy numbers for a defined fluorescence signal [23]. In addition to *in vitro *synthesis of cRNA standards as performed in this study, the standard curves are commonly obtained using plasmid clones containing the cDNA of the gene of interest as the template by quantitative PCR. Compared with plasmid clones, cRNA standards was applicable for measuring transcripts from any gene of interest and can be reverse-transcribed and amplified with RNA from samples in a parallel procedure using identical primer pairs.

For accurate quantification of PCR products, the slope of the standard curve obtained with 10-fold dilutions should approach -3.3 in theory, but a slope from -3.1 to -3.6 was acceptable in practice. Furthermore, the corresponding correlation coefficient should be >0.95 [24]. The correlation coefficient and the reaction efficiencies of the standard curve constructed in this study were 0.995 and 103.2%, respectively. The slope from the linear equation was -3.247, which was close to the theoretical slope of -3.3, maintaining linearity for at least six orders of magnitude. The maximum CV with the mean Ct values of 29.25 was 2.63%, demonstrating good reproducibility of the assay.

To evaluate the specificity of PCR products amplified, a melting curve analysis should be performed by detecting primer-dimers and non-specific products. The specificity of PCR-amplified products was determined by only a single peak visible in the melting peak profile [24,25]. In this study, there was no evidence of non-specific amplification in melting curve of each sample, indicating a high specificity of the SYBR Green I RT-PCR assay for the detection of BVDV in cell culture.

The one-step SYBR Green I RT-PCR assay in this study was 10-fold more sensitive than the conventional RT-PCR assay. It could discriminate most of the copy numbers of BVDV from 10^-1 ^TCID_50 _to 10^-4 ^TCID_50 _in their adjacent virus titers and detect the virus RNA as low as 10^-5 ^TCID_50 _which were unable to be differentiated or detected by conventional RT-PCR. Compared with conventional PCR assay, the SYBR Green I real-time PCR assay was a more efficient method with a lower detection limit and higher sensitivity [26]. It has also been demonstrated to be more rapid, sensitive and reliable than virus isolation by traditional cell culture [20].

The single-tube detection assay and one-step SYBR Green I real-time PCR assay were respectively developed for detecting the virus [27,28]. The SYBR Green I RT-PCR assay established in this study was one-step real-time RT-PCR assay in one tube, which reduces the risk of contamination of samples and shares the advantages of both one-tube and one-step.

In conclusion, this study developed an one-step SYBR Green I real-time quantitative RT-PCR assay for detecting BVDV RNA in cell culture, which has high reproducibility and specificity, and is more sensitive than conventional RT-PCR. This method has developmental potentials in diagnosing and screening the animals infected with BVDV. It may also be applied to evaluate candidate agents against HCV using BVDV cell culture model.

## Competing interests

The authors declare that they have no competing interests.

## Authors' contributions

NZ conceived and performed the experiments. ZL involved in conceiving and designing the experiments and writing the manuscript. QH contributed reagents and materials and involved in revising the manuscript. JQ and JC involved in designing the study and revising the manuscript. GZ, ZL, SL and NL involved in performing the experiments and data analysis. All authors read and approved the final manuscript.
